# Supplementary material for: Protective Effects of Chestnut (Castanea crenata) Inner Shell Extract in Macrophage-Driven Emphysematous Lesion Induced by Cigarette Smoke Condensate
Source: Nutrients. 2023 Jan 4;15(2):253. doi: 10.3390/nu15020253 (PMC9867500; doi:10.3390/nu15020253)

Figure S1. Neutrophil counts in bronchoalveolar lavage fluids. NC; mice instilled with vehicle + vehicle p.o., CSC; mice instilled with CSC + vehicle p.o., CSC+CIE100 and CSC+CIE300; mice instilled with CSC + CIE p.o., 100 or 300 mg/kg/day, respectively. Values: means ± SD (n = 6). Significance: $^{\#\#}p < 0.01$ vs NC.

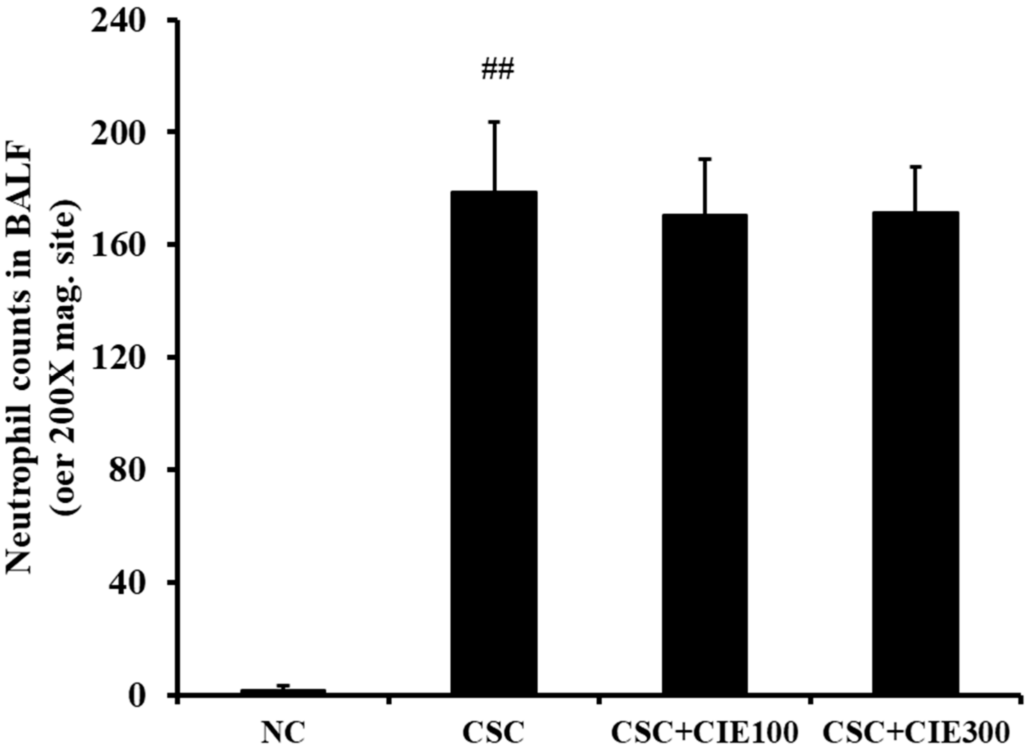

Figure S2. Whole blot and the molecular marker of MMP-9 shown in Figure 3.

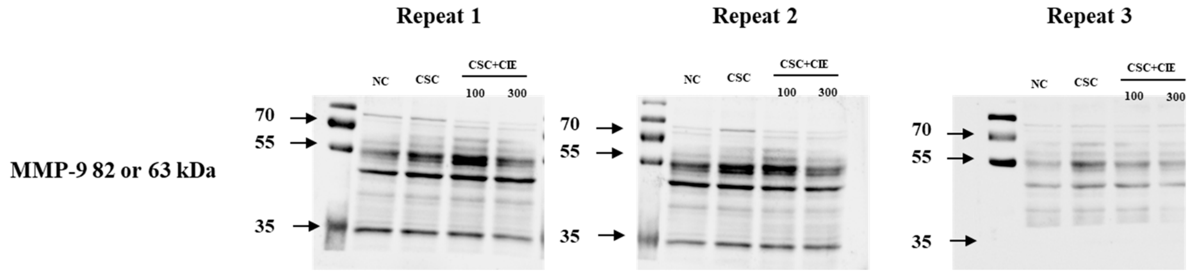

Figure S3. Whole blot and the molecular marker of each protein shown in Figure 4.

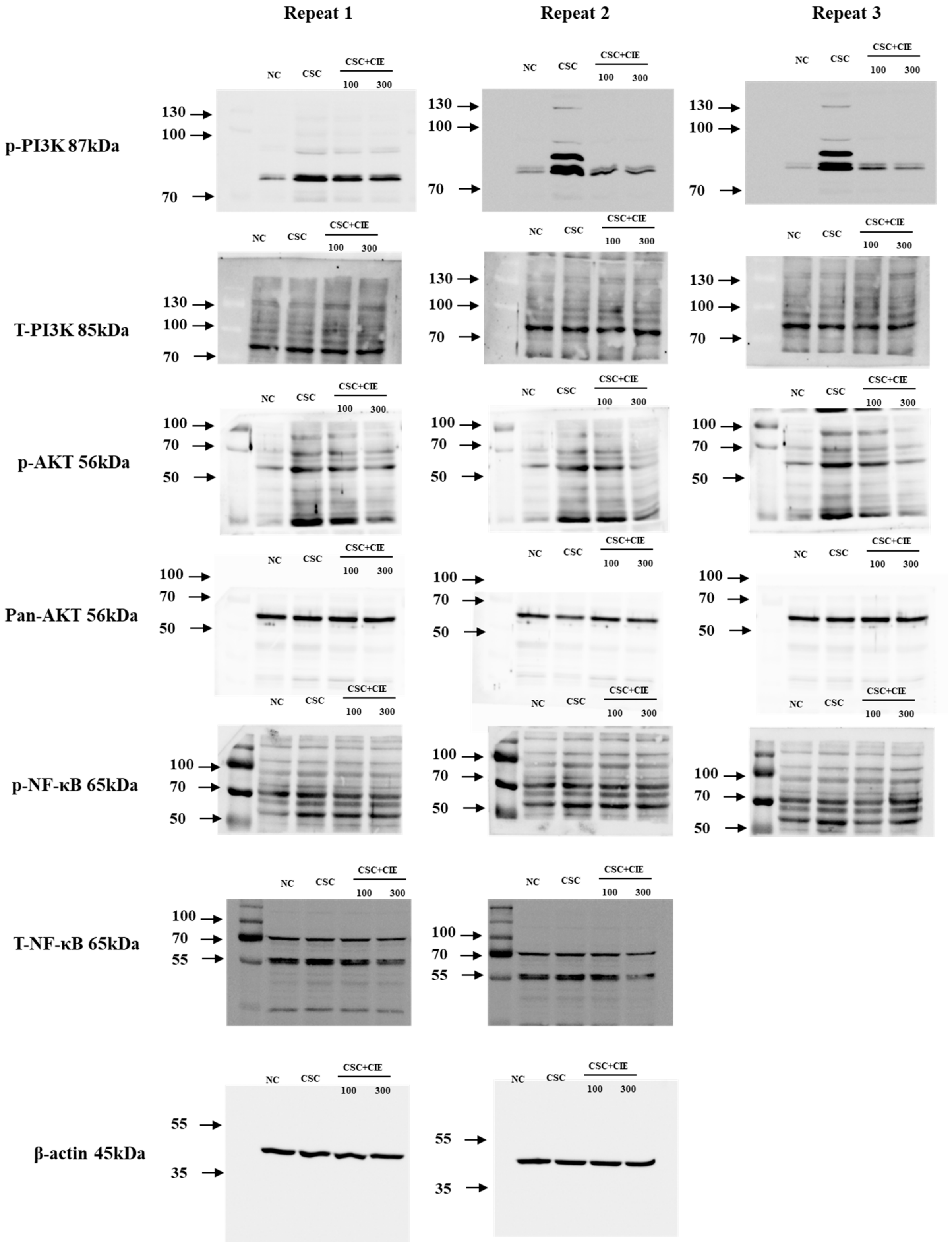

Figure S4. Whole blot and the molecular marker of each protein shown in Figure 6.

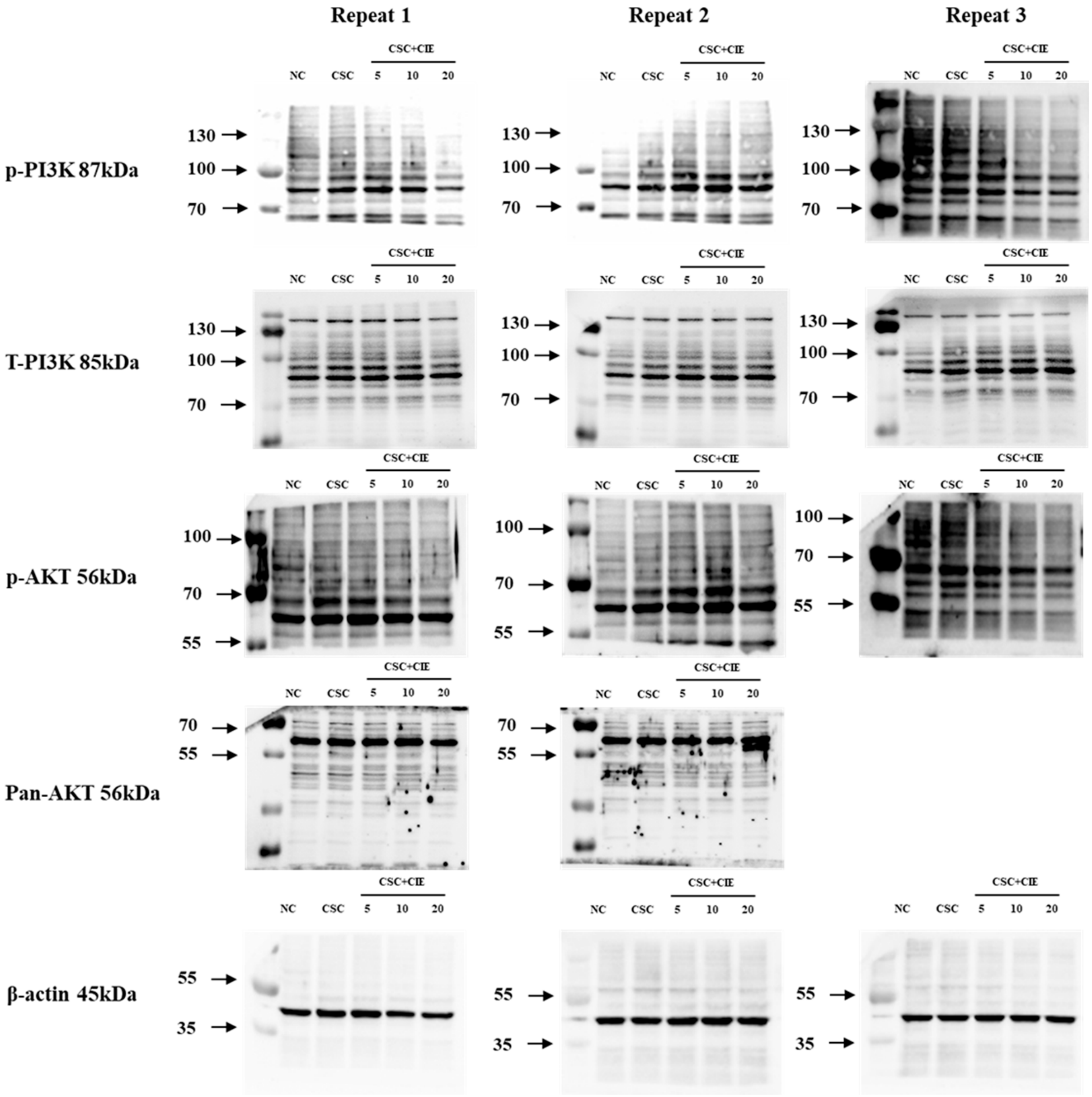

Figure S5. Whole blot and the molecular marker of each protein shown in Figure 7.

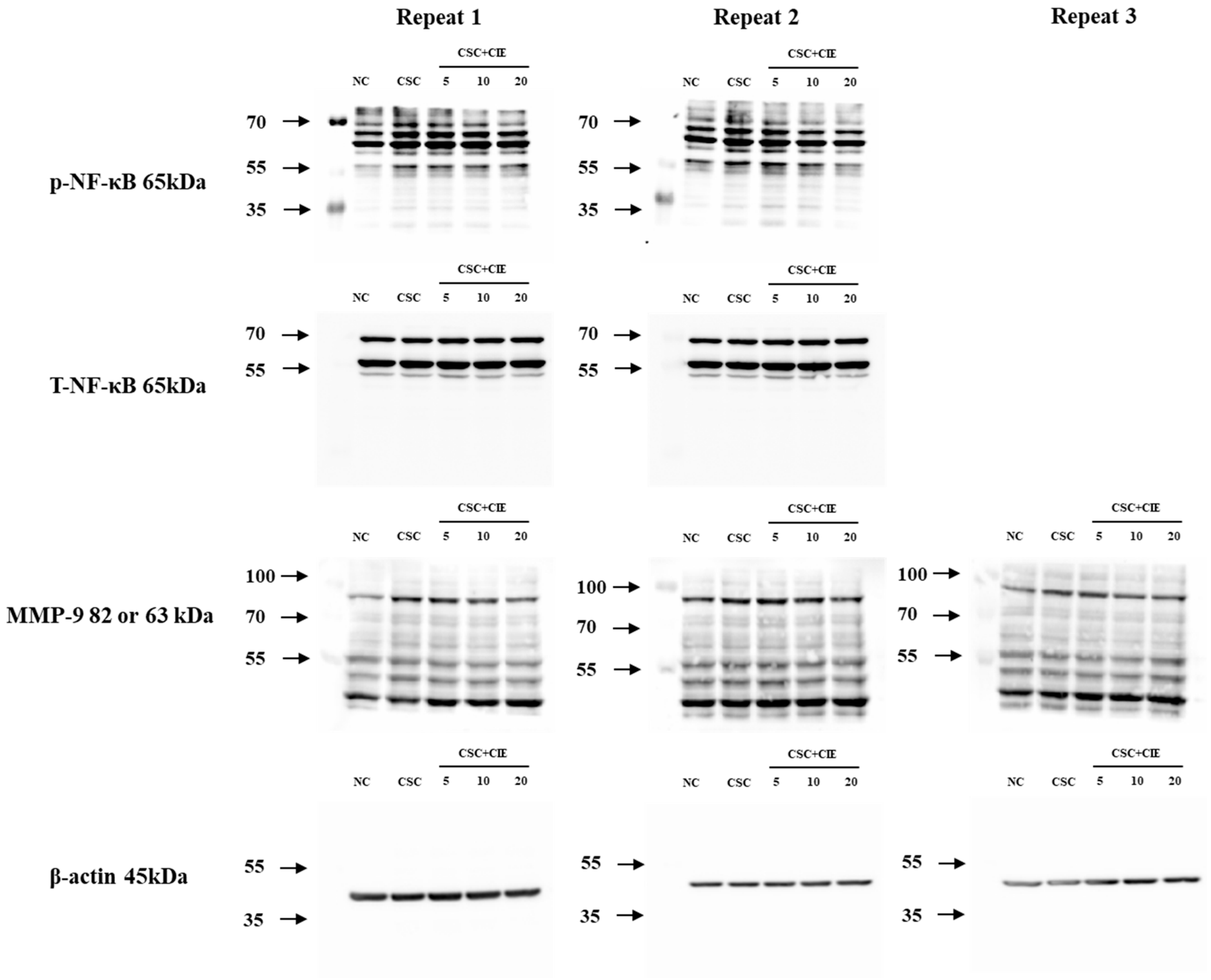

Supplement: Supplementary file 1 [file nutrients-15-00253-s001.zip › nutrients-2081710-Supplementary Material.pdf]
